# Targeting PDK1 for Chemosensitization of Cancer Cells

**DOI:** 10.3390/cancers9100140

**Published:** 2017-10-24

**Authors:** Aikaterini Emmanouilidi, Marco Falasca

**Affiliations:** Metabolic Signaling Group, School of Biomedical Sciences, Curtin Health Innovation Research Institute, Curtin University, Perth 6102, Western Australia, Australia; a.emmanouilidi@postgrad.curtin.edu.au

**Keywords:** PDK1, phosphoinositides, PI3K, chemotherapy, chemoresistance

## Abstract

Despite the rapid development in the field of oncology, cancer remains the second cause of mortality worldwide, with the number of new cases expected to more than double in the coming years. Chemotherapy is widely used to decelerate or stop tumour development in combination with surgery or radiation therapy when appropriate, and in many cases this improves the symptomatology of the disease. Unfortunately though, chemotherapy is not applicable to all patients and even when it is, there are many cases where a successful initial treatment period is followed by chemotherapeutic drug resistance. This is caused by a number of reasons, ranging from the genetic background of the patient (innate resistance) to the formation of tumour-initiating cells (acquired resistance). In this review, we discuss the potential role of PDK1 in the development of chemoresistance in different types of malignancy, and the design and application of potent inhibitors which can promote chemosensitization.

## 1. Introduction

### 1.1. The Journey to PDK1 Discovery

The 3-phosphoinositide-dependent protein kinase 1 (PDK1) was identified in 1997, in a strenuous attempt by a number of research groups to elucidate the insulin signaling pathway. Around 1990 it had been observed that within seconds of the interaction of insulin with its receptor, the phosphoinositide 3-kinases Class 1A (PI3K Class 1A) would be recruited to the plasma membrane and would mediate the generation of phosphatidylinositol (3,4,5)-trisphosphate (PtdIns(3,4,5)*P*_3_) from phosphatidylinositol (4,5)-bisphosphate (PtdIns(4,5)*P*_2_). During the same period, protein kinase B (PKB/Akt) was discovered by a number of research groups and it was shown that within just one-minute following cell stimulation, insulin could trigger the activation of Akt in a PI3K-dependent manner. The phosphorylation motif of Akt present in its substrates was determined and many proteins were shown to possess it and get activated following insulin stimulation, however the theory that another kinase—part of the PI3K pathway was responsible for these phosphorylations was still being investigated. In 1996, the interaction between Akt and PtdIns(3,4,5)*P*_3_ came to light, yet the activation mechanism of the first remained unknown. Studies showed that following insulin stimulation, Akt underwent phosphorylation at Thr308 (activation loop) and Ser473 (hydrophobic motif), with nonsynonymous substitution of either of the two leading to more than 85% reduction of Akt activation, and that membrane recruitment was required for phosphorylation of both residues (reviewed in [1]). It was not until 1997 that a protein responsible for Akt phosphorylation at Thr308 was detected, purified and cloned. This was the 3-phosphoinositide-dependent protein kinase 1 (PDK1), a protein which interacted with PtdIns(3,4,5)*P*_3_ and PtdIns(4,5)*P*_2_ through its pleckstrin homology (PH) domain and exhibited a dramatic increase in its ability to activate Akt in the presence of these phosphoinositides. The necessity of these phosphoinositides lies in the facts that they change the configuration of Akt in a way that the Thr308 residue is much more approachable for PDK1, and that they enable the simultaneous localization of both PDK1 and Akt in the membrane, allowing for the first to phosphorylate the latter [2,3]. PDK1 was later shown to phosphorylate more members of the AGC (cAMP-dependent, cGMP-dependent and protein kinase C) kinase family, including the serum-and glucocorticoid-induced protein kinases (SGKs), the ribosomal protein S6 kinase beta-1 (S6K1) and the protein kinase C (PKC) (reviewed in [1]). The next step was to elucidate the mechanism behind the regulation of PDK1, which possesses five constantly phosphorylated Ser residues that remain unaffected by the action of insulin. One of them, Ser241, is a prerequisite for PDK1 activity and it seems like the protein gets auto-phosphorylated on that residue [4]. After many observations it was proposed that PDK1 is constitutively active and that phosphoinositides are responsible for converting its substrate into a form susceptible to phosphorylation by PDK1 [1].

### 1.2. Physiological Roles of PDK1 and Its Correlation with Malignancies

The physiological importance of PDK1 was clear in knockout mice which exhibited lethality at the second embryonic week (E9.5) [5]. Lethality at the embryonic stage was also shown in knock-in models with mutations of either the PDK1-interacting fragment (PIF) pocket [6] or the PH domain [7]. To overcome the issue of embryonic lethality, any interventions performed in the mouse models would now be tissue-specific. Muscle and liver PDK1^−/−^ mice would only be viable for up to 4 months, and would suffer heart failure, and glucose intolerance followed by liver failure, respectively [8,9,10]. Pancreas PDK1^−/−^ mice would be viable but diabetic [11], whereas the models created with conditional knock-in tissue-specific strategies would exhibit different phenotypes ranging from hyperinsulinemia, glucose intolerance and smaller body size, depending on the mutation type [12,13]. The studies performed on hypomorphic mice revealed a number of roles of PDK1, including the management of gastric acid secretion levels [14] and the stimulation of the Na^+^/H^+^ exchanger via the serum- and glucocorticoid-inducible kinase 1 (SGK1) and the consequent regulation of the transport of electrolytes in the intestine [15]. Decreased PDK1 activity leads to the distortion of amino acid transport in the jejunum and increased amino acid excretion in the urine, implying defective renal reabsorption and decreased transport of amino acids in the kidneys and the intestine.

The PDK1 protein is a member of the AGC kinases and it is encoded by the PDPK1 gene, located at 16p13.3. This specific locus amplification has been correlated with poor survival prognosis in breast cancer, and has also been detected in lung and prostate cancer [16,17]. In breast cancer in particular, PDK1 is the means by which upstream lesions such as *PTEN*, *PIK3CA* and *ERBB2* boost their signal output and reach to Akt, rendering the cells resistant to PI3K pathway inhibitors [18]. Increasing evidence suggests that PDK1 plays a pivotal role in cell migration [19], while it is able to control cell motility via ROCK1 and has a significant effect in three-dimensional (3D) environments [20]. It is interesting that whereas in monolayer cell culture the downregulation of PDK1 seems to have no effect, its inhibition in 3D environments decreases cancer cell invasion in an Akt-dependent manner, suggesting that this could be a target to counteract cancer invasiveness (reviewed in [19,21,22]). The PI3K pathway is often deregulated in malignancies and exhibits high PtdIns(3,4,5)P3 levels. Being part of the pathway PDK1 is also implicated in cancer, in the majority of the cases though it is the overexpression that leads to pathologic conditions, rather than mutations. For instance, increase in gene copy number and protein overexpression have been reported in breast cancer and acute myeloid leukaemia, among other malignancies [23]. During melanoma initiation, PDK1 is the intermediate for the PKC regulation by the tissue inhibitor of metalloproteinase-1 (TIMP1), and in later stages of progression and metastasis, it promotes resistance to *anoikis* [24]. In non-small cell lung cancer, glutamine shortage leads to the PDK1/Akt axis activation, which in turn promotes metastasis [25]. Tissue sample analysis of patients with hepatocellular carcinoma who underwent surgical resection but where not submitted to any other type of therapy, revealed that the PDK1 mRNA levels were the most potent factor regarding time to recurrence (TTR) prediction and were also correlated with decreased overall survival rate [26].

Due to the great matter of attention drawn to PI3K and Akt as the major molecules in order to target many aspects of cancer, the role of PDK1 in cancer has been overlooked. However, the ability of the latter to act independently of them two, has revamped the focus on this molecule and its pharmacological targeting [7]. Indeed, the ability of PDK1 to drive tumorigenesis in an Akt-independent manner has been recently demonstrated. More specifically, Vasudevan et al. demonstrated that in the context of existing *PIK3CA* mutations, Akt activity is deficient and SGK3 serves as the main PDK1 effector [27]. Mutations in *PIK3CA* seem to be a late event in tumorigenesis, and they mostly result in increase of the kinase activity, allowing for *PIK3CA* to act as an oncogene. Two significant hotspot mutations of *PIK3CA* occur in the helical domain in exon 9 (E545K) and the kinase domain in exon 20 (H1047R) [28,29]. Cells harbouring such *PIK3CA* mutations exhibit higher levels of phosphorylated PDK1, and these specific mutations seem to partially control the recruitment of PDK1 on the membrane. This can be attributed to a function that is not related to the kinase activity of PIK3CA; for instance maintenance of adaptor-proteins that facilitate membrane recruitment of PDK1 irrespectively of the PH domain. The *PIK3CA* mutant cells highly depend on SGK3 for their survival, and it is possible that PI3K exerts its signaling through the endosomes, where SGK3 localizes and subsequently engages PDK1. The study concluded that tumours with *PIK3CA* mutations exhibit Akt dependency when there is manifestation of defective feedback regulation or phosphate and tensin homolog (PTEN) signaling, whereas they exert Akt- independent signaling and they recruit SGK3 when such deficiencies are absent [27]. In addition to this, another study showed that although breast cancer cells growth is independent of PDK1, their ability for tumour initiation in vitro relies on it, regardless of their PIK3CA burden. *p53* and *K-RAS* mutated cells are more prone to inhibition of PDK1 than *PIK3CA* mutated ones, but their growth in 3D conditions remains uninfluenced by Akt inhibition, in contrast to the ones with mutated *PIK3CA*. Kinase activity of the molecule is a prerequisite for 3D growth, but the same does not apply for the PH domain, thus leading to the conclusion that Akt is not an effector molecule in the tumorigenic activity of PDK1. In line with this, constitutively active Akt was not able to redeem for PDK1 loss in regards to 3D growth and its chemical or genetic inhibition did not alter the PDK1 hyperactivation outcome [30].

Lately, many cases have been reported where PDK1 is directly or indirectly implicated in chemoresistance, with its inhibition resulting in re-sensitization of cancer cells to chemotherapeutic agents, and this is going to be the topic of discussion in the following sections.

## 2. PDK1 in Chemoresistance

### 2.1. Ovarian Cancer

Ovarian cancer has a high mortality among women and the PI3K pathway has been shown to be the main pathway exhibiting aberrant expression and deregulation, affecting the progression of the disease [31]. The importance of PDK1 in ovarian cancer is underscored by an immunohistological analysis of the expression of the peroxisome proliferator-activated receptor *β* (PPAR*β*) and PDK1 in healthy ovary tissue, benign tumours and many malignant subtypes. In this analysis, although PPAR*β* staining was positive in healthy and benign conditions, PDK1 was absent in 90% of the healthy tissue specimens. Only one healthy specimen and less than half of the benign ones exhibited weak PDK1 expression, whereas all malignant as well as borderline tumours were stained positively. These findings strengthen the notion of the oncogenic capacity of PDK1 in ovarian cancer [32]. Very recently, Moxley et al. demonstrated that PDK1 is implicated in ovarian cancer via an alternative splicing product of the Ron receptor tyrosine kinase, named short-form Ron (sfRon) [33]. This protein is absent from normal ovarian tissue but is strongly expressed in malignant ovarian tumours, especially in one of the most lethal types, the high-grade serous one. Overexpression of sfRon in OVCAR3 cells resulted in a more aggressive phenotype both in vitro and in vivo, induced epithelial-to-mesenchymal transition (EMT) reflected by vimentin and N-cadherin upregulation and E-cadherin reduction, and instigated the PDK1 signaling pathway, which was depicted by increase of pSer241-PDK1 and pThr308-/pSer473 AKT [33]. In agreement to the group’s previous studies, this model revealed that sfRon was dependent on PI3K signaling to induce EMT, one of the most crucial steps of the pathway being plasma membrane recruitment of PDK1, and this PI3K signaling effects were AKT/mTOR-independent [34]. Notably, PDK1 has previously been held accountable for EMT initiation in gallbladder cancer [35]. The fact that OVCAR3-sfRon cells had a very firm PDK1 expression compared to the absence of the protein’s expression in control OVCAR3 cells, reflects the importance of PDK1 in driving aggressiveness in ways that are not correlated to AKT, and that focus should be given on testing inhibitors of PDK1 alone or in combination with Ron inhibitors, especially in this type of ovarian cancer [33].

Epithelial ovarian carcinoma (EOC), one of the most dismal malignancies among women, exhibits chemoresistance via increase of phosphorylated Akt levels, which is achieved by stabilization of the PDK1 protein by the collagen type XI aplha1 (COL11A1). COL11A1-PDK1 binding sequesters the latter and protects it from degradation via the ubiquitin (Ub)-proteasome pathway (UPP), which is normally induced by paclitaxel and cisplatin, thus conferring resistance to chemotherapy [36]. COL11A1 is physiologically known to be essential for the cartilage, since its presence allows for differentiation of the chondrocytes and formation of the collagen fibrils [37]. Its mRNA levels are significantly elevated in chemoresistant EOC cell lines, compared to chemosensitive ones and is found to play a role in paclitaxel and cisplatin resistance. Studies on A2780CP70 (cisplatin-resistant) and A2780 (cisplatin-naïve) cells indicated that both paclitaxel and cisplatin- but not doxorubicin or gemcitabine, induced COL11A1 levels dose- and time-dependently, whereas no similar activity was observed with the naïve cell line. In parallel, the genetic modification of *COL11A1* had a clear impact on the half maximal inhibitory concentration (IC_50_) of the chemotherapeutic drugs, as its knockdown increased the responsiveness of the A2780CP70 cells to cisplatin and paclitaxel, and its overexpression in the naïve cell line decreased it. Further experimental work revealed that both drugs triggered the interaction between the *COL11A1* promoter and the CCAAT/Enhancer Binding Protein Beta (c/EBPβ) [36]. Interestingly, the Akt pathway increases the c/EBPβ expression [38], and chemical inhibition of the phosphatidylinositol 3-kinase (PI3K) inhibits the aforementioned interaction and promotes chemosensitivity in A2780 cells. Notably, genetic downregulation of *COL11A1* was found to attenuate the expression of the Akt pathway components such as phospho-Akt and PDK1, which led to the notion that COL11A1 increased the stability of PDK1 protein. Indeed, *COL11A1* silencing promoted PDK1 degradation through the proteasome pathway and immunoprecipitation assays indicated that both cisplatin and paclitaxel strengthened the COL11A1-PDK1 binding, whereas PDK1 knockdown in COL11A1- overexpressing cells was able to reduce c/EBPβ and phospho-Akt levels [36].

In the context of sensitivity to chemotherapeutic drugs, a specific PDK1 inhibitor termed 2-*O*-benzyl-myo-inositol 1,3,4,5,6-pentakisphosphate (2-*O*-Bn-InsP_5_), had an additive effect in combination with rapamycin and was able to enhance the effect of paclitaxel on SKOV-3 cancer cells. This compound inhibited PDK1 with an IC_50_ of 26.5 nM and reduced phosphoThr308-Akt both in vitro and in vivo, while it was also capable of in vitro impeding mTOR in the low micromolar range, raising the question of whether it could be further investigated as a dual inhibitor [39].

A very interesting example of the redundant pathways adapted by cancer cells to promote their survival in different aspects was very recently demonstrated by Gocher et al. Previous studies have mentioned that Akt can be activated by Ca^2+^/calmodulin-dependent protein kinase kinase 2 (CAMKK2) in ovarian, prostate and embryonic kidney cells, however Gocher et al. addressed the question of whether ovarian cancer resistance to platinum was a consequence of a combined action of CAMKK and the canonical PI3K pathway. Indeed, CAMKK2 was shown to regulate the phosphorylation of Akt at both the threonine and serine residues in ovarian cancer cells, and combined downregulation of the CAMKK2 and PDK1 proteins had comparable effects on Akt phosphorylation, as did pharmacological inhibition of CAMKK2 and PI3K. This provides significant information that CAMKK2 is able to sustain Akt phosphorylation when the PI3K pathway is inhibited. There could be a number of explanations for the simultaneous existence of these two pathways for the regulation of Akt. One possibility could be that the low availability of growth factors in the hypoxic tumour environment was insufficient for complete activation of the PI3K pathway, so CAMKK2 was an alternative way to compensate and sustain tumour growth [40]. Alternatively, it is possible that growth factors exploit the calcium-dependent pathway to modulate the Akt activation levels, as in the case of phospholipase Cε (PLCε), explained in [41].

It is worth mentioning that in cell lines derived from serous epithelial ovarian cancer, Yes-associated protein 1 (YAP) acts as an oncogene implicated in various cancer characteristics. Transfection of cells with pSer147-YAP led to significant boost of their colony formation capacity, in contrast to cells transfected with wild type protein or a mutant with a defective WW domain and at the same time allowed these cells to surpass contact inhibition in monolayer culture and increased their invasion and migration potential in transwell assays. Moreover, pSer147-YAP shielded ovarian cancer cells against Taxol and cisplatin treatment, and in ovarian cancer patients there was an association between nuclear sequestration of the protein and reduced progression free survival (PFS) [42]. PDK1 plays a very crucial and fine-balanced role in pSer147-YAP related activities, which will be extensively discussed in Section 3.

### 2.2. Breast Cancer

Breast cancer is the most common cancer type affecting women worldwide and amplification of the *PDK1* genetic locus is correlated with low survival rates. One fifth of breast tumours exhibit *PDK1* amplification, with increase of the phosphorylated protein (pSer241-PDK1) levels being a frequent event in this malignancy, which is also present in more than three fourths of metastatic cases. Both pharmacological and genetic inhibition of PDK1 in vitro have demonstrated its significance for all steps of breast cancer progression, and in vivo experiments display its role in growth and metastasis [43]. Anti-oestrogen therapies apply on more than half of breast cancer cases, due to the expression of oestrogen receptor α (ERα) which automatically categorizes them as oestrogen signaling dependent. Therapy with tamoxifen is among the most widely used, however almost half of the patients with an early onset, as well as all patients with metastasis, develop resistance to it. In 2009, Iorns et al. performed both siRNA and compound screening to identify a molecular pathway as a good candidate for enhancing sensitivity to tamoxifen, with all findings pointing to the PDK1 pathway. More specifically, the nucleoside derivative triciribine was found to be a potent molecule- inhibitor of PDK1 and was used in further studies which suggested that counteracting the pathway in question could in fact sensitize breast cancer cells not only to tamoxifen but to also to other types of endocrine therapy such as fulvestrant (ICI 182780). Moreover, genetic silencing of *PDK1* in combination with tamoxifen result in G1 cell cycle arrest and upregulation of p21Cip1. This was the first study to demonstrate the suitability of PDK1 as a target towards restoration of sensitivity to tamoxifen [44]. Soon after this, the aforementioned compound 2-*O*-Bn-InsP_5_, was also shown to reduce the survival of breast cancer cells in vitro in combination with tamoxifen, in a more effective manner than the two drugs did separately. The same was observed when it was administered in conjugation with curcumin and paclitaxel [39]. As for PDK1, there is a possibility that it is able to tune signaling cascades initiated by the oestrogen receptor (ER), thanks to its control over the forkhead box O (Foxo) (discussed in [45]). 

In addition to these, OSU-03012, a derivative of celecoxib, a cyclooxygenase-2 (COX2) inhibitor, which accounts for PDK1 but not COX2 inhibition [46], managed to sensitize breast cancer cell lines to tamoxifen therapy independently of their ER status and the ER downstream pathway. OSU-03012 was able to counteract the tamoxifen-induced phosphorylation of Akt in MCF-7 cells, and to some extent in MDA-MB-231 cells, and restore cell responsiveness to therapy. It is also notable that daily treatment of MDA-MB-231 tumours in nude mice with a combination of tamoxifen and OSU-03012 resulted in 50% suppression of tumour growth [47]. This compound exerts its PDK1 inhibitory activity (IC_50_ of 5 μM) by competing the adenosine triphosphate (ATP) binding and in parallel it hampers p70^S6K^ and Akt activation. Further details, as well as chemical structure and properties can be found in [46].

It is interesting that despite the fact that PDK1 is overexpressed in a number of related cancer cell lines and patients’ samples [48,49], the current norm for treatment focuses on different components of the pathway, such as the epidermal growth factor receptor-2, and there is limited ongoing research on the evaluation of PDK1 as target. Meanwhile, elevated phospho-PDK1 was correlated to insusceptibility to gemcitabine-mediated cell death, compared to elevated phospho- Akt, and *PDK1* silencing rendered MCF-7 cells more responsive to the drug, than did Akt silencing. These observations highlight the suitability of PDK1 as a focus point in order to restore chemosensitivity, especially in regard to gemcitabine, in breast cancer cells [50]. The enhanced gemcitabine resistance conferred by PDK1 in contrast to Akt1, can be partially attributed to the fact that PDK1 acts relatively earlier in the pathway and its activation affects a wider range of molecules, including protein kinase Cα (PKCα) which has been reported to be overexpressed concomitantly with PDK1 and is implicated in gemcitabine resistance [51,52]. In addition to that, there is only one phosphorylation site within the activation loop that is required for PDK1 to become active, and the protein can be also found in a constitutively activated form. On the contrary, complete Akt1 activation is more complicated and requires for a series of phosphorylations to take place [50]. All together, these findings underscore the action of PDK1 via an Akt-independent mechanism.

A recent study concluded that PDK1 plays a role in triple negative breast cancer (TNBC) chemoresistance. More specifically, this type of cancer exhibits aberrant levels of fatty acid-binding protein 5 (FABP5) which through retinoic acid (RA) delivery is known to enhance the peroxisome proliferator-activated receptor β/δ (PPARβ/δ) at the transcriptional level and activate the vascular endothelial growth factor-A (VEGF-A) and PDK1. Curcumin was able to render TNBC cells sensitive to retinoid therapy, by restricting the FABP5 expression and reducing RA-induced *PDK1* transcription; with the exact mechanism connecting RA and PDK1 yet to be elucidated [53]. The effects of curcumin on breast, as well as pancreatic and prostate cancer cells have been shown to be enhanced when curcumin is combined with 2-*O*-Bn-InsP_5_, a specific PDK1 inhibitor [39].

### 2.3. Acute Myeloid Leukaemia

Acute myeloid leukaemia (AML) is another type of cancer with dismal prognosis due to lack of responsiveness to chemotherapy and common relapse, accountable for which are leukaemia stem cells (LSCs). Upon leukaemia patients’ chemotherapy, a small LSCs population that exhibited resilience and managed to survive, starts to proliferate and turns into the dominant population, paving the way for disease relapse [54]. The characteristics of LSCs such as quiescence, low division rate and resistance to therapy, state them an intractable target; yet a recent study indicated PDK1 as a LSCs survival regulator and thus an appealing target for therapy [55]. Almost half of AML sufferers with poor prognosis exhibit overexpression of PDK1 [56] and hematopoietic malignancies including AML often exhibit inactivation of PTEN and Akt activation, which is strongly correlated with poor prognosis. This Akt activation is in part due to aberrant regulation of other signaling pathways such as Bcl-2-associated death promoter (BAD) and *p53* [57,58,59,60]. As abovementioned, recent studies revealed that *PDK1* deletion not only had a positive effect on the lifespan of an MLL-AF9 mouse model and reduced LSCs incidence following secondary transplantation, but also significantly enhanced *p53* and *Bax* expression [55], both of which are known for their pro-apoptotic properties [61]. In the clinical setting, tumours with high levels of *p53* are considered more responsive to chemotherapy, whilst *Bax* downregulation is linked to both limited drug sensitivity and decreased survival [62,63]. Another interesting observation of the same study was that *PDK1* deletion was coincident with lower *Stat5* levels, raising the notion that this might be the main LSCs maintenance pathway [55], since Stat5 is constitutively active in many types of leukaemia [64].

### 2.4. PDK1 and Chemoresistance in Multiple Types of Cancer

Metadherin (MTDH) is an oncogene thought to affect many cancer related pathways such as PI3K/AKT and Wnt, and its downregulation was shown to increase the chemosensitivity of many tumours such as prostate and breast cancer to 5-fluorouracil (5-FU), paclitaxel and doxorubicin; the exact mechanism though had remained unknown. A recent study came to unravel this mechanism of action, demonstrating that MTDH protects cancer cells by interfering with the cell cycle checkpoints and initiating pro-survival cascades. MTDH downregulation resulted in caspase-3 and -8 mediated endometrial cancer cell death, following treatment with tumour necrosis factor-α-related apoptosis-inducing ligand (TRAIL) and the LBH589 histone deacetylase (HDAC) inhibitor. MTDH depletion coincides with a decline in PDK1 phosphorylation which renders the cell prone to apoptosis via the invigoration of Bim expression. A protein of interest in this case is galectin-1, which has a strong presence in a number of tumour types and is implicated in the PI3K pathway. Knockdown of this protein was shown to avert PtdIns(3,4,5)*P*_3_ increase following stimulation with insulin growth factor 1 (IGF1) in glioblastoma cells, and this study denoted that MTDH-induced galectin-1 increase might result in an increase of PtdIns(3,4,5)*P*_3_ levels and activation of the PI3K cascade. Overall, these data exhibit that MTDH constitutes a target for more effective chemotherapy, with PDK1 and Bim playing key role in this procedure [65].

Whilst the constitutively active PDK1 does not undergo any further activation derived from mitogenic signals [3], its activity is modulated by interactions with other proteins-modulators; for instance the 14-3-3 protein which reduces its activity [66], the heat shock protein 90 (Hsp90) which protects it from the activity of the proteasome [67], and the tumour suppressor candidate 4 (TUSC4), which was found to be implicated in cancer cells chemosensitivity. More specifically, TUSC4 negatively regulates the PDK1 downstream cascade, as it is able to form a ternary complex with Src and PDK1 and attenuate the latter’s Src-induced tyrosine sites phosphorylation; thus, hampering the S6K and Akt activation. Cells modulated to express TUSC4 were found to be more responsive to chemotherapeutic drugs including doxorubicin, taxol, cDDP and VP-16, than their control counterparts. This underscores the significance of PDK1 inhibition in overcoming resistance to anticancer drugs [68].

**Figure 1:**
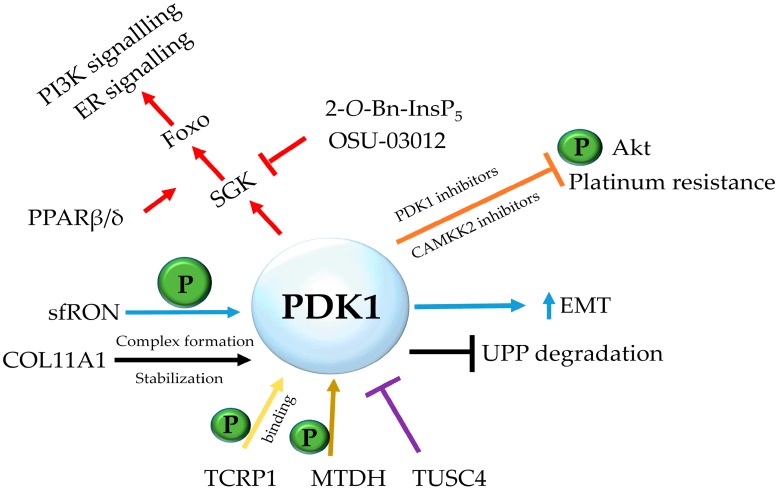
Major signaling pathways in chemoresistance and PDK1 implication. PDK1 is implicated in a number of signaling pathways and cancers. Complex formation with COL11A1 results in PDK1 stabilization and resistance to ubiquitin (Ub)-proteasome pathway degradation in epithelial ovarian carcinoma, its instigation by sfRON leads to EMT in high-grade serous ovarian cancer, whereas PDK1 inhibition combined with CAMKK2 inhibition leads to reduction of phospho-Akt levels and decreased platinum resistance in ovarian cancer. Sensitization of breast cancer cells to drugs such as tamoxifen and paclitaxel is correlated with the effect and sustaining of PI3K and ER signaling through SGK1/3 and Foxo1/3 isoforms, where components such as PPARβ/δ have a stimulating effect, and PDK1 inhibitors such as 2-*O*-Bn-InsP_5_ and OSU-03012 have proven to be effective. In other types of cancer, proteins such as MTDH and TCRP1 are shown to directly or indirectly activate PDK1, and the tumour suppressor TUSC4 is shown to form a complex with it and negatively regulate the signaling cascade.

PDK1 can be also implicated in chemoresistance through another PDK1-binding protein, the tongue cancer resistance-related protein-1 (TCRP1), the levels of which are found to be increased in a number of cancers including pancreatic and ovarian ones [69]. TCRP1 is correlated with cisplatin resistance in lung cancer [70] and oral squamous cell carcinoma resistance to radiation [71]. Aberrant expression of this protein induces phosphorylation of PDK1 in a PTEN and PI3K-independent manner; TCRP1 binds to PDK1 via two amino-acid sequences (T109-A124 and R93-S107) and it is speculated to promote 14-3-3/PDK1 dissociation, leaving PDK1 in a monomer configuration that not only has the ability of auto-phosphorylation, but can also phosphorylate its downstream target, Akt. Nonetheless, further studies need to be carried out concerning the TCRP1-mediated PDK1 phosphorylation. TCRP1 was shown to induce cell transformation via PDK1 activation, and chemical (OSU-03012) or genetic downregulation of the latter reversed this phenomenon [69]. A summary of the aforementioned pathways where PDK1 is implicated can be found in Figure 1.

## 3. PDK1 Oncogenic Signaling in Chemoresistance: Beyond AKT

### 3.1. PDK1-PLK1-MYC Axis

**Figure 2:**
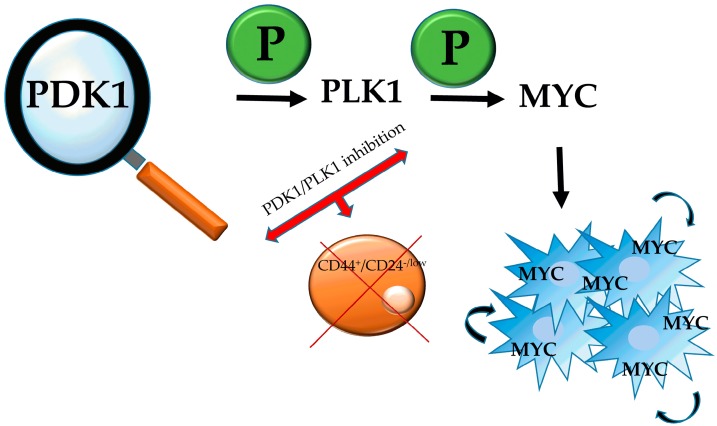
The PDK1-PLK1-MYC axis. PDK1, via PLK1 phosphorylation and subsequent MYC phosphorylation, results in the sequestration of the latter in cancer cells. MYC is known for its ability to promote self-renewal of cancer cells as well as stem cells, and therefore inhibition of either of the first two components of the axis leads to depletion of cancer stem-like cells.

Recently, there have been a number of reports showing that in some cancer cases PDK1 acts independently of the PI3K pathway to exert its oncogenic properties [27,72,73]. PDK1 has been shown to act through another route, that of Polo-like kinase 1 (PLK1)-MYC [74]. *MYC* is a well-studied oncogene, with its respective protein being involved in the ability of cancer cells, as well as stem cells, to self-renew [75], and was lately proved to be PDK1-dependent in order to induce HEK cells transformation. PDK1 triggers the phosphorylation of PLK1, which is also upregulated in many cancers, and the latter interacts with MYC, phosphorylates it and results in its accumulation in cancer cells (Figure 2). MYC-driven breast cancer is shown to be more responsive to PDK1/PLK1 inhibitors than is MYC-independent one, and taking into consideration that a MYC inhibitor is not currently available in the clinic, targeting the PDK1-PLK1-MYC axis reveals a new potential therapeutic approach against MYC-induced cancers. It is also likely that chemoresistance will be affected as well, since inhibition of either PDK1 or PLK1 resulted in depletion of the CD44^+^/CD24^−/low^ stem cell-like populations in the MDA-MB-231 cell line, a phenomenon not observed when the PI3K-AKT pathway was impaired [74].

### 3.2. PDK1-YAP/Hippo Pathway Axis

**Figure 3:**
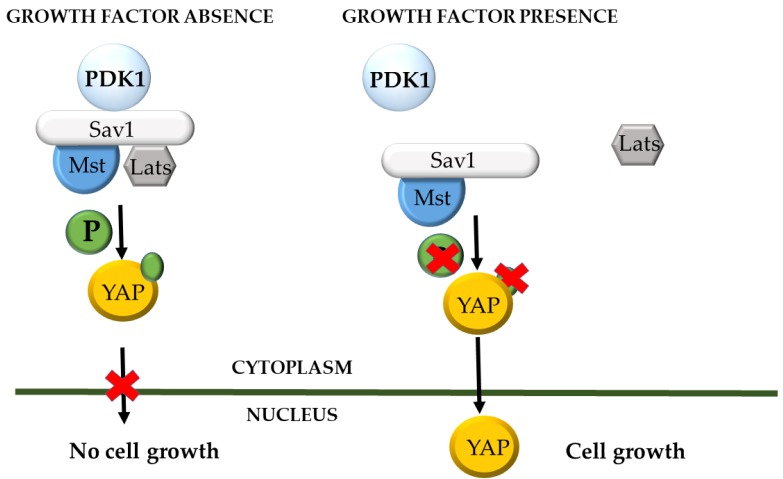
PDK1 and the Salvador/Warts/Hippo (SWH) pathway. When epidermal growth factor receptor (EGFR) signaling is active, PDK1 is recruited to the cell membrane and the core components of the HIPPO pathway are not forming a complex, thus YAP protein is in its inactive form and can insert the nucleus and act as a transcription factor for growth-promoting genes. On the opposite, in the absence of growth stimuli, a cytosolic complex is formed between the core proteins of the HIPPO pathway and PDK1, which phosphorylates YAP and renders it in the cytoplasm, excluding its nuclear entry.

The Salvador/Warts/Hippo (SWH) pathway, alternatively named Hippo signaling pathway, was initially discovered and studied in Drosophila, where it coordinates organ size. The main core of the cascade is comprised of the tumour suppressor proteins Warts (Wts), Salvador (Sav), Hippo (Hpo) and Mob-as-tumour-suppressor (Mats) and mutations leading to loss of function of any of them results in an increased proliferation- or hippopotamus-like phenotype. The respective mammalian homologues are the Lats1/Lats2, Sav1 or WW45, Mst1/Mst2 and MOBKL1A/1B, which shape a conserved cassette that responds upon high cell density signals, and inactivates YAP by phosphorylation. More specifically, the kinase complex formed by Sav1 and Mst/HIPPO phosphorylates Lats kinase, which targets the Ser127-YAP and results in its restriction to the cytoplasmic compartment. Inactivation of the pathway leads in YAP nuclear translocation, where is acts as a transcriptional activator for genes related with proliferation (reviewed in [76]). This activation is mediated by TEA domain transcription factors (TEADs) and it has been demonstrated that the promoter of the connective tissue growth factor (CTGF) gene is bound by the YAP-TEAD1 complex in MCF10A and NIH-3T3 cells. In cancer cells overexpressing YAP, knockdown of either of the aforementioned complex components had a major impact on the CTGF mRNA levels, and knockdown of the CTGF itself abrogated cell growth [77]. In MCF10A serum starved cells which have reached the point of contact-inhibition, YAP is not detected in the nucleus, however this phenomenon is quickly reversed by epidermal growth factor (EGF) treatment. EGF acts via the Hippo pathway by inhibiting Lats, and therefore reduces Ser127 phosphorylation of YAP, promoting its recruitment in the nucleus and allowing it to exert its transcriptional activity. Treatments with inhibitors of PI3K, PDK1 and its downstream effectors revealed that it was only the first two kinases that were involved in YAP phosphorylation and nuclear accumulation, therefore it is the PI3K-PDK1 signal that links EGFR with the SWH pathway. Further experiments showed that signals unrelated to EGFR, such as horse serum or LPA, could initiate the PI3K cascade and cause nuclear accumulation of YAP, suggesting that signals upstream of this pathway are able to inhibit the Hippo cascade. PDK1 forms a complex with the core SWH pathway proteins (Sav1, Mst, Lats), which can be disrupted following EGF treatment. This dissociation can be prevented with the use of PDK1 and PI3K inhibitors. Analysis of the complex showed that since PDK1 lacks the domains needed to directly interact with all the components of the HIPPO pathway, Sav1 is the mediator molecule. Sav1 relates to PDK1 via its 145–162 residues and allows for the PDK1-Lats and PDK1-Mst interactions via its WW and SARAH domains, respectively. The proposed model connecting PDK1, YAP and EGF signaling was that in growth factor signaling absence, PDK1 is found in the cytoplasm in the form of a complex with the Hippo components, leading to phosphorylation and cytoplasmic retention of YAP. Cell stimulation with growth factors leads to plasma membrane recruitment of PDK1 and subsequent disruption of the complex, resulting to Lats inactivation, pSer147-YAP levels reduction and accumulation of the dephosphorylated protein in the nucleus, where it acts as a transcriptional activator for growth controlling genes [78] (Figure 3).

### 3.3. PDK1-SGK Axis

Aberrant expression of the PI3K/AKT/mTOR pathway is a frequent phenomenon in breast cancer and it is the result of a PIK3CA mutation, which corresponds to the PI3K p110 catalytic subunit, and more specifically to isoform α. Regardless of the development of specific PI3Kα inhibitors, not all tumours are responsive and the molecular mechanisms sustaining this resistance need to be defined. Even upon complete PI3K/AKT inhibition, there is a remaining mTORC1 activity which allows cancer cells to overcome PI3Kα- inhibitors treatment [79,80,81,82,83]. The classic pathway by which PI3K triggers mechanistic target of rapamycin complex 1 (mTORC1) activity, is initiated by G-protein-coupled receptors (GPCRs) or RTKs. As mentioned in previous sections, AKT is phosphorylated by PDK1 but can only exert its full activity following phosphorylation by mTORC2. Once fully active, AKT can inhibit the tuberous sclerosis complex (TSC) by phosphorylating it in four serine and one threonine residues (S1132, S1130, S981, S939 and T1462). The TSC acts as a GTPase activating protein (GAP) for the Ras homolog enriched in brain (Rheb) GTPase, and its inhibition results in activation of the latter, which in turn activates mTORC1. Consequently, AKT phosphorylates the inhibitory subunit 40-kDa proline-rich (PRAS40) causing it to dissociate from the complex, rendering mTORC1 accessible to substrates (extensively reviewed in [84]). Yet, this seems to not be the case in tumours resistant to PI3Kα inhibition, as recent research has shown that PDK1 is a new player actively underpinning this resistance. Experiments using breast cancer cell lines carrying PI3Kα mutations and therefore resistant to the specific PI3Kα- inhibitor BYL719, revealed that the observed residual mTORC1 activity was PtdIns(3,4,5)*P*_3_- and thus AKT- independent, and stands in need of both PIF-binding pocket and kinase activity of PDK1. Results from simultaneous PI3Kα and PDK1 inhibition using BYL719 and GSK2334470 suggested that resistant cells were acquiring a transcriptional activity dependent on forkhead box proteins O (FOXOs), and more specifically on FOXO3 which showed robust nuclear sequestration upon dual treatment [85]. While in the presence of growth stimuli the 14-3-3 proteins restrain FOXO proteins in the cytoplasm, their cessation causes dephosphorylation and nuclear transport of the proteins in question, leading to expression of pro-apoptotic genes [86]. Despite the fact that FOXO1/3 have been identified as targets of AKT, its complete inhibition does not seem to result in the expected FOXO3 nuclear translocation in BYL719 resistant cells and so, researchers focused on the detection of a protein that would comprise a member of the AGC kinases, associate with the PDK1 PIF-binding pocket and be dependent on its catalytic activity, while it would concomitantly affect mTORC1 and FOXOs activity in an AKT unbiased manner. Transcriptomic analysis of a number of breast cancer cell lines with different BYL719-resistance status pointed out to SGK1, since the phosphorylation levels of its target N-Myc Downstream Regulated 1 (phosphoNDRG1) were significantly elevated compared to total protein in resistant cell lines [85]. Although SGK1 and pNDRG1 levels vary accordingly in vivo, and AKT has been shown to target NDRG1 in vitro [87,88] as well as in vivo in the case of mouse models with PDK1 K465E knock-in mutation [89], the significance of SGK1 in this mechanism was confirmed by the fact that pNDRG1 levels were not affected by BYL719 treatment in resistant cells. On the contrary, it was the combination of both the GSK2334470 and BYL719 inhibitors that sufficed to reduce this phosphorylation. The outcomes of this study showed that pharmacological targeting of SGK1 was a realistic and achievable objective and that complete inhibition of mTORC1 requires dual targeting of SGK1 and AKT. Apart from these, another novelty was the discovery that in fact it is the phosphorylation of TSC2 by SGK1 that is responsible for this residual mTORC1 activation. It should be kept in mind that although AKT and SGK1 may both be under the control of PDK1 and mTORC2, what makes a big difference is that AKT contains a PH domain which renders it dependent on plasma membrane recruitment, whereas SGK1 can be active even when PtdIns(3,4,5)*P*_3_ is unavailable. The partial reduction in SGK1 activity that follows PI3Kα inhibition in resistant cell lines, can be explained by the fact that PtdIns(3,4,5)*P*_3_ can affect mTORC2 in a mammalian stress-activated protein kinase interacting protein 1 (mSIN1)-dependent manner. There also exist other intracellular mTORC2 pools with ambivalent localization and different dependency on growth factors status, which can explain this observation [85].

Apart from SGK1, SGK3 also holds a key role in melanoma resistance to PI3K/Akt inhibition [90]. *BRAF* mutations, and especially BRAF^V600E^, are an early and very frequent event in melanoma. This specific mutation promotes PTEN silencing to sustain the progress from a benign to a malignant state. It was recently shown that independently of their Akt and PTEN status, melanoma cells are prone to PI3K or BRAF^V600E^ inhibition, and even more sensitive to a dual inhibition. Noticeably, mTORC1/2 inhibition was able to apprehend cell proliferation comparably to BRAF^V600E^ or PI3K inhibition. It was further demonstrated that PI3K and BRAF^V600E^ were able to control mTORC1 activity in an AKT-independent manner [91]. Following these studies, another group showed that SGK3 together with PDK1 are implicated in melanomas harbouring BRAF mutations and wild type PTEN, which in fact account for more than half the cases of melanomas occurrence. SGK3, being a substrate of PDK1, acts as its mediator, and inhibition of either of the two kinases leads to cell cycle arrest at G1 phase. Simultaneous inhibition of PDK1 and PI3K/mTOR or the proteasome exhibits synergism and has a higher impact on melanoma proliferation [90]. Due to the important role of Akt in the maintenance of cancer cells, it would be a natural consequence that following extended Akt or PI3K Class I inhibition, the cells would urge to recompense for that loss. Indeed, a very efficient strategy undertaken by malignant cells is the upregulation of SGK3, which has a high level of overlapping targets with Akt and can be activated by hVps34 in a PI3K Class I-independent manner. hVps34 acts by producing PtdIns(3)P, to which SGK3 can bind via its PX domain and be subsequently phosphorylated and activated by PDK1, and it is worth mentioning that SGK1 and SGK2 lack a PX domain and depend on PI3K for their activation. TSC2 phosphorylation and the resulting SGK3 and mTORC1 activation can comprise an Akt-independent cascade responsible for the occurrence of chemoresistance [92].

### 3.4. Inhibitors of PDK1

**Figure 4:**
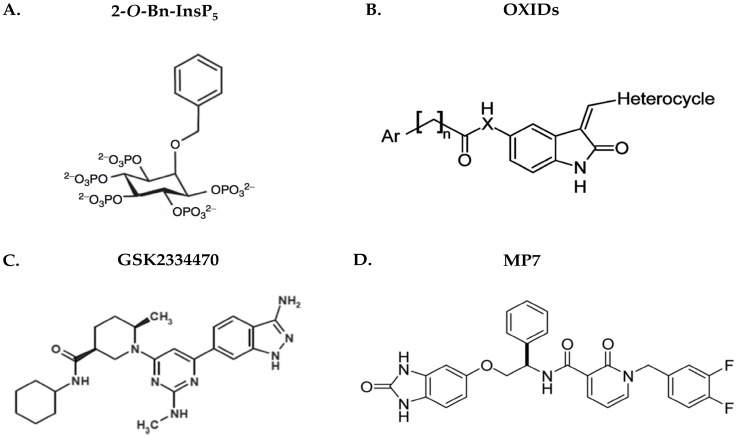
Structures of commonly used PDK1 inhibitors. (**A**) 2-*O*-Bn-InsP_5_ (M. Falasca Laboratory); (**B**) OXIDs (S. Rapposelli Laboratory); (**C**) GSK2334470 (GlaxoSmithKline); (**D**) MP7 (Merck).

Since its discovery in 1997, PDK1 has attracted the interest of the research community and a number of patents have been established by different companies and institutions, including Merck & Co. (Kenilworth, NJ, USA), Boehringer Ingelheim International GmbH (Ingelheim am Rhein, Germany), GlaxoSmithKline (Brentford, UK), Novartis International AG (Basel, Switzerland), Biogen Idec Inc. (Cambridge, MA, USA), Sunesis Pharmaceuticals (South San Francisco, CA, USA), Wyeth (now Pfizer Inc., Manhattan, NY, USA), Ohio State University, University of London and University of Bath [93]. It is interesting that in a commentary article, Alessi—a leading scientist in the PDK1 research field—and Peifer specifically emphasize that “just because PDK1 is not on many researchers’ radars does not mean it is not a key anti-cancer target” [45]. It is true that the research community focuses on different elements of the pathway such as mTOR and Akt, nevertheless inhibition of PDK1 specifically has an advantage over them, due to the fact that as a key regulator it controls an impressive number of other kinases including Akt, SGK and ribosomal S6K. Moreover, it is now well-established that PDK1 activates a unique signaling pathway distinct from the canonical Akt- and PI3K-dependent pathways. The fact that the protein exists in a single isoform makes it an even more attractive target for inhibition in malignant conditions [94]. An extensive overview on PDK1 inhibitors until 2008, explains in detail how the ATP binding site of PDK1 has been used as a scaffold to create the majority of the inhibitors and analyses the mechanism of action of all the known categories, from small molecule inhibitors (bisindolmaleimides, LY333531, LY317615, UCN-01, substituted thieno[3,2-*c*]pyridine-7-carboxamides, indolinones, pyridinonyl-PDK1 inhibitors, *N*- phenylpyrimidin-2-amines, 4-heterocycloalkyl-2-aminopyrimidines, diazepinones) to tetracyclic imidazophenanthrenones (imidazo[4,5-*c*]quinolones, pyrrole derivatives, quinazolines, celecoxib derivatives, 4-aryl-7-azaindoles, 3,5-diaryl-7-azaindoles, pyrrolo[2,3-*d*]pyrimidines, pyrazole[1,5-*α*]pyrimidines, triazolo[1,5-*α*]pyrimidines, pyrazolylbenzimidazoles, indazoles, dibenzo[c,f][2,7]-naphthyridines), 3-hydroxyanthranilic acid and activators/modulators of PDK1 [94]. Apart from the aforementioned PDK1 inhibitors, one more compound has been recently identified, the inositol 1,3,4,5,6-pentakisphosphate (InsP_5_), termed 2-*O*-Bn-InsP_5_ (Figure 4A), which exerts anti-tumour and pro-apoptotic functions, while at the same time renders cancer cells prone to chemotherapeutic drugs such as tamoxifen and curcumin. Its mechanism of action is proposed to take place through binding of the PH domain of PDK1, which results in retention of the latter in the cytosol, thus hampering phosphorylation and activation of Thr308 of Akt [39,95]. The antineoplastic effects of InsP_5_ were validated in vivo, where administration of the drug in an ovarian cancer xenograft resulted in growth inhibition akin to cisplatin [96]. Moreover, derivatives of 2-oxindole (OXIDs) (Figure 4B), have been shown to disrupt the PDK1/Akt pathway and comprise good candidates for non-small cell lung cancer treatment [97], and these results initiated studies for the synthesis and assessment of new derivatives within the same family, which gave rise to promising candidates for the targeting of glioblastoma multiforme (GBM) [98].

Recently, a very potent PDK1 inhibitor termed GSK2334470 (Figure 4C) has been developed by GlaxoSmithKline and has shown remarkable specificity towards PDK1 (IC_50_ ≈ 10 nM), when tested among almost a hundred protein kinases together with members of the AGC-kinase family. This compound was shown to inhibit the phosphorylation of the SGK T-loop and the NDRG1 protein as well as the endogenous S6K1 phosphorylation on the Thr229 of the T-loop, following IGF1 stimulation. Though it was less efficient in decreasing Akt activity compared to PI3K inhibitors, GSK2334470 had a great impact on components such as GSK3 and FoxO. This can be explained by the fact that both PDK1 and Akt contain PH domains which enable them to bind PtdIns(3,4,5)*P3* and co-localize on the surface of the membrane, remarkably increasing chances of interaction. Thus, even the slightest fraction of PDK1 escaping GSK2334470 inhibition would be sufficient to activate Akt, which is in agreement with the observation that cytosolic Akt lacking PH domain (ΔPH-Akt) was more potently inhibited that the full-length protein. Another notion is that PDK1 anchored to the plasma membrane is less accessible to the drug than the cytosolic one. It is important to remember that Akt controls a number of functions crucial for the cells’ survival, and its inhibition could have severe side effects. Since there are also other pathways driving tumorigenesis, which are independent of Akt but require SGK, the evaluation of a compound like GSK2334470 would be of great clinical importance [99]. A structure-based explanation of the moderate effect of this inhibitor on Akt phosphorylation levels is the reverse allosteric effect, where the PDK1-substrate interaction mediated by the PIF-pocket, is regulated by the ATP- binding site (reviewed in [100]). The binding of a compound to the PIF-pocket would probably stabilize the protein conformation regardless of its activation status at that point in time, and it is speculated to have higher specificity than ATP- binding site targeted molecules. This was demonstrated in vitro, where such molecules were shown to be able to inhibit S6K phosphorylation and signaling, but did not affect Akt (reviewed in [101,102]). Last but not least, following observations in conditional knock-in animal models expressing a mutated PDK1 PH domain, where Akt was exhibiting partial activation [13], a later model proposed that PDK1 is able to interact with pSer473-Akt via the PIF-pocket, and can therefore be independent of PtdIns(3,4,5)*P3* binding. In fact, distortion of the PIF-dependent mechanism was shown to render Akt more susceptible to PDK1 inhibitors such as GSK2334470 [103].

Currently, preclinical testing of GSK2334470 in a number of cancer cases is showing encouraging results. Advanced breast cancer (ER^+^) is initially treated with CDK4/6 inhibitors, such as palbociclib, and anti-estrogens, but a number of patients fail to benefit from this therapy due to acquired or de novo resistance. The use of GSK2334470 was shown to act synergistically with CDK4/6 inhibitors to promote apoptosis and inhibit cell proliferation, while at the same time it was able to restore chemosensitivity in cells resistant to palbociclib [104]. The same compound has been shown to negatively affect the proliferation of multiple myeloma (MM) cell lines and overcome their resistance to dexamethasone, leaving the non-malignant cells unaffected [105]. Nevertheless, it was found that the responsiveness of the cells to this inhibitor was proportionally correlated to the expression status of PTEN, supporting the idea that following loss of PTEN, sole PDK1 inhibition does not suffice to hamper cancer progression [73]. In this case, although pSer241-PDK1 and pSer2448-mTOR levels were decreased following GSK2334470 administration, cells exhibiting low PTEN expression maintained the pSer473-AKT and pSer2481-mTOR levels. Restoration of PTEN levels resulted in decreased levels of these phosphoproteins and therefore inhibition of their activity, depicting a novel perspective in the mechanisms of MM resistance to PDK1 inhibition. The mTOR cascade has been proposed as an appropriate target in MM, but the existing major barrier is that inhibition of mTORC1/C2 causes increase in phosphorylation of IGFR-1 in MM cells, and blocks apoptosis. Interestingly, GSK2334470 pro-apoptotic effects cannot be reversed by IGFR-1 upregulation and when combined with PP242 (an inhibitor of mTORC1/C2) it is cytotoxic for both chemosensitive and chemoresistant MM cells. Collectively, this evidence emphasizes the pro-apoptotic effects of the combination of PDK1 and mTOR inhibition in MM, independently of the PTEN expression levels [105].

Recently, Sunesis Pharmaceuticals has disclosed data regarding two promising PDK1 inhibitors, SNS-229 and SNS-510, which seem to be effective in haematological malignancies resistant to Akt and PI3K inhibition. In contrast with GSK2334470, these inhibitors act via intervention in the PIF-pocket and disruption of substrate binding, and have shown auspicious in vitro and in vivo preliminary results, and superiority over GSK2334470 [106,107].

PDK1 inhibition has also been shown to be effective as part of a multitargeted therapy regime. In regards to GBM, combination of MP7 (Figure 4D) (characterized in [108]) and alisertib which are PDK1 and Aurora Kinase A (AurA) inhibitors respectively, exhibited higher potential of reducing both the proliferation of GBM cells and their respective tumorspheres, than sole administration of each agent. A recently discovered OXID-pyridonyl derivative named SA16 was shown to be a dual PDK1/AurA pathway inhibitor which successfully reduces cell proliferation and invasiveness, while inducing the differentiation and subsequent apoptosis of GMB stem-like cells, therefore reducing their population and pointing towards a solution to GBM chemoresistance [109].

## 4. Conclusions

Due to the crucial role PDK1 holds regarding the cell physiology, it would be reasonable to argue that apart from the medical benefit, PDK1 inhibition would have multiple side effects. This protein is a crucial regulator of signaling pathways so important, that any deregulation would have detrimental effects. Apprehension would gradually lead to type II diabetes [110], whereas overstimulation would result in excessive proliferation and malignant conditions [111]. Nevertheless, animal studies showed that mice with only 10% of the *PDPK1* function exhibited a normal phenotype and when crossed with PTEN^+/−^ mice, tumour formation capacity was disrupted [112]. Interestingly, there is currently a number of PI3K inhibitors used in the clinical setting, which have not fulfilled the initial expectations though. This could be due to inadequate effect on the final target or to the fact that more molecules of the same pathway need to be co-targeted. This is where PDK1 inhibitors can be introduced and have a synergistic effect, counteracting invasion, metastasis and chemoresistance [113]. In breast cancer, it was recently shown that PDK1 inhibition was able to re-sensitize *PIK3CA*-mutant cells to PI3Kα inhibitors through suppression of SGK1 and reduction of mTORC1 activity [85]. The PI3K pathway holds an important role in embryonic stem cell fate, with the main effector being PDK1 [114], and in addition to the implication of the latter in cancer stem cells maintenance mentioned in previous sections, it has been shown that dual inhibition of checkpoint kinase 1 (CHK1) and PDK1 effectively eliminates glioblastoma stem-like cells responsible for resistance to therapy and tumour recurrence [115]. Given the crucial role of cancer stem cells in the course of malignancies and their impact on the efficiency of therapies and the overall survival of the patients, application of the existing knowledge to target PDK1 and continuous efforts for the discovery of new molecules-inhibitors, are of utmost importance. 

The significance of PDK1 lies in the fact that in terms of targeted therapy, it can replace undruggable molecules such as KRAS. *KRAS* is mutated in approximately 90% of pancreatic cancer cases but all efforts for its direct targeting have proven unfruitful. Since PDK1 comprises the most crucial effector downstream of KRAS, it is the best alternative for targeted therapy in this type of cancer. More specifically, in pancreatic cancer, *KRAS* mutations result in a constitutively active protein which upregulates the PI3K/Akt cascade; the suppression of which is not feasible due to loss of *PTEN* in earlier stages of the disease (reviewed in [116]). Interestingly, Eser et al. demonstrated that KRAS downstream effectors exhibit tissue-specificity and provided evidence that PI3K/PDK1 is a suitable target in pancreatic ductal adenocarcinoma (PDAC) [117]. Additionally, it seems that a specific micro-RNA, miR-375 is the missing link connecting these two proteins and its expression is significantly different in normal versus pancreatic cancer tissues, correlating also with PDK1 expression levels (reviewed in [116]). Moreover, emerging evidence is pointing towards a role of PDK1 in the tumour microenvironment. PDK1 is necessary for the T-cell receptor to convey its signal, for the maturation of the T-cell, migration of endothelial cells and neutrophils chemotaxis among others, however it was shown that it is pivotal for tumour angiogenesis, which is one of the hallmarks of cancer, as well as proliferation and progression (reviewed in [118]).

Due to the key role of PDK1 in a number of cellular functions, it would be reasonable to argue whether apart from the delivery of beneficial effects, PDK1 inhibition could cause serious side effects to the subjects in clinical trials. The answer to this question comes via a plethora of preclinical studies in a variety of animal models for diverse diseases. Early studies in a transgenic adenocarcinoma of the mouse prostate (TRAMP) model showed that following treatment with OSU-03012, metastasis occurrence was minimized, as well as lobe proliferation, and in fact all prostate lobes exhibited a weight reduction. Nevertheless, other compound-related in vivo effects were observed such as weight loss which could not be counteracted by food intake, and compromise of the Type II skeletal myofibers [119]. On the contrary, OSU-03012 was able to cross the blood-brain barrier and exhibit good drug tolerability and effective tumor growth inhibition in schwannoma xenograft mouse models [120]. In prion and Alzheimer’s disease studies, different mouse models treated with the BX912 PDK1 inhibitor, acquired ameliorated cognitive skills, social behavior and memory, and scored higher in multiple tasks, comparing to control mice. Further benefits included improved motor function, prolonged survival and decreased levels of PrP^Sc^ in the brain. However, one model suffered BX912 toxicity at day 350- therefore mitigating the beneficial effects of the inhibitor regarding Alzheimer’s disease, but not undermining the overall outcomes of the study [121]. *In vivo* studies have shown that GSK2334470 in combination with PP242, an mTORC1/C2 inhibitor, was able to significantly abrogate tumor growth in xenograft multiple myeloma mouse models, comparing to sole administration of the inhibitors [105]. Preclinical studies of SNS-229 and SNS-510 in CD/1 mice, carried out by Sunesis Pharmaceuticals, have shown satisfying oral bioavailability (>90%) and pharmacokinetic properties (retrieved from https://www.sunesis.com/pap_pdk1.php), and no side effects were reported from either of the studies. In addition to these, nude mice treated with BX-320 exhibited no observable side effects, and the number of treated animals that died was equal to the control ones, with deaths being attributed to the dosing regimen rather than toxicity of the inhibitor [122]. Overall, it can be concluded that PDK1 inhibition does not result in any serious side effects and can be well-tolerated, a fact that can pave the way to Phase I clinical trials.

For the reason that Akt plays a multifaceted role in normal and pathologic cellular conditions, it has been the centre of attention for therapeutic strategies development, overlooking other members of the AGC family. PDK1 has been an emerging and promising target for anti-cancer therapies, which through both an Akt-dependent and independent manner is implicated in many aspects of carcinogenesis. Application of the most potent inhibitors on tumour models will reveal an integrated picture of the PDK1 abrogation potential in carcinogenesis (discussed in [21]). In parallel with this, PDK1 could serve well in the field of cancer biomarker discovery, and more specifically act as a pharmacodynamic (PD) biomarker, in order to measure the biological activity of a protein-target, following treatment with an inhibitor- candidate. For instance, during kinase drug discovery, the levels of phosphorylation sites on the kinases- targets are measured and quantified, instead of the levels of the phosphorylation levels of the protein- substrates, for the reason that the latter can be affected by pathway cross-talk and compensatory mechanisms. In the case of PDK1, the Thr^513^ and Ser^410^ phospho-residues constitute targets for PDK1 inhibitor molecules and their dephosphorylation levels reflect the potency of the inhibitor. In fact, PDK1 inhibitors type I, which compete for the ATP-binding site were shown to have an impact on these sites, but not on pSer^241^ which resides in the T-loop, as was shown by immunoaffinity precipitation- mass spectrometry (IAP-MS). Supporting these findings, the PDK1 *bona fide* readouts such as pRSK^Ser221^ and pAKT^Thr308^ were not representative of the PDK1 inhibition in prostate cancer cells [123]. There is currently little literature information regarding the two aforementioned PDK1 phosphorylation sites and their effect on the kinase activity, and further studies would shed more light on this matter [4,124]. PhosphoSer^241^ PDK1 has also been identified and served as a candidate PD biomarker for the prediction of the efficacy of a DGF-out PDK1 inhibitor named compound 7 [108].

To conclude, we have here presented a thorough literature review assessing the value of PDK1 in chemosensitization of cancer cells, analyzed Akt-dependent and independent pathways, and reported the available inhibitors up to date, while providing information on the preclinical validation of these inhibitors and the studies outcome, as well as the potential role of PDK1 as a pharmacodynamic biomarker.

## Abbreviations

3D	Three dimensional
5-FU	5-fluorouracil
AML	acute myeloid leukaemia
ATP	adenosine triphosphate
AurA	Aurora Kinase A
BAD	Bcl-2-associated death promoter
c/EBPβ	CCAAT/Enhancer Binding Protein Beta
CAMKK2	Ca^2+^/calmodulin-dependent protein kinase kinase 2
cDDP	cis-diamminedichloroplatinum(II)/cisplatin/cisplatinum
COL11A1	collagen type XI aplha1
COX2	cyclooxygenase-2
CTGF	connective tissue growth factor
EGF	epidermal growth factor
EMT	epithelial-to-mesenchymal transition
EOC	epithelial ovarian carcinoma
ERα	oestrogen receptor α
FABP5	fatty acid-binding protein 5
Foxo	forkhead box O
GAP	GTPase activating protein
GBM	glioblastoma multiforme
GPCR	G-protein-coupled receptor
HDAC	histone deacetylase
Hpo	Hippo
Hsp90	heat shock protein 90
IC_50_	half maximal inhibitory concentration
IGF1	insulin growth factor 1
K-RAS	Kirsten Rat Sarcoma Viral Oncogene Homolog
Lats	Large Tumor Suppressor Kinase 1
LPA	lysophosphatidic acid
LSC	leukaemia stem cells
Mats	Mob-as-tumour-suppressor
MLL-AF9	mixed lineage leukemia-ALL1- fused gene from chromosome 9 protein
MM	Multiple myeloma
MOBKL1A/1B	Mps one binder kinase activator-like 1A
mRNA	messenger ribonucleic acid
mSIN1	mammalian stress-activated protein kinase interacting protein 1
MTDH	metadherin
mTOR	mammalian/mechanistic target of rapamycin
MYC	v-myc myelocytomatosis viral oncogene homolog (avian)
NDRG1	N-Myc Downstream Regulated 1
nM	nanomolar
p21Cip1	cyclin-dependent kinase inhibitor 1
PDAC	pancreatic ductal adenocarcinoma
PDK1	3-phosphoinositide-dependent protein kinase 1
PH	pleckstrin homology
PI3K	phosphoinositide 3-kinase
PIF	PDK1-interacting fragment
PKB/Akt	protein kinase B
PKC	protein kinase C
PLCε	phospholipase Cε
PLK1	Polo-like kinase 1
PPAR *β*	peroxisome proliferator-activated receptor *β*
PRAS40	inhibitory subunit 40-kDa proline-rich
PtdIns(3,4,5)*P*_3_	phosphatidylinositol (3,4,5)-trisphosphate
PtdIns(4,5)*P*_2_	phosphatidylinositol (4,5)-bisphosphate
PTEN	phosphate and tensin homolog
RA	retinoic acid
RTK	receptor tyrosine kinase
Rheb	Ras homolog enriched in brain
S6K1	Ribosomal protein S6 kinase beta-1
Sav	Salvador
Ser	serine
sfRon	short-form Ron
SGK	Serum and glucocorticoid-induced protein kinase
Src	V-Src Avian Sarcoma (Schmidt-RuppinA-2) Viral Oncogene
SWH	Salvador/Warts/Hippo
TCRP1	tongue cancer resistance-related protein-1
TEADs	TEA domain transcription factors
Thr	threonine
TIMP1	tissue inhibitor of metalloproteinase-1
TNBC	triple negative breast cancer
TRAIL	tumour necrosis factor-α-related apoptosis-inducing ligand
TSC	tuberous sclerosis complex
TUSC4	tumour suppressor candidate 4
UPP	ubiquitin (Ub)-proteasome pathway
VEGF-A	vascular endothelial growth factor-A
VP-16	Etoposide Phosphate
Wts	Warts
YAP	Yes-associated protein 1
μΜ	micromolar
